# Supplementary material for: Structural basis of RECQL5-induced RNA polymerase II transcription braking and subsequent reactivation
Source: Nat Struct Mol Biol. 2025 Jul 7;32(9):1731–40. doi: 10.1038/s41594-025-01586-6 (PMC12440807; doi:10.1038/s41594-025-01586-6)
Supplement: Supplementary file 1 — Reporting Summary [file 41594_2025_1586_MOESM1_ESM.pdf]

nature portfolio

Corresponding author(s): Suyang Zhang

Last updated by author(s): Apr 8, 2025

Reporting Summary

Nature Portfolio wishes to improve the reproducibility of the work that we publish. This form provides structure for consistency and transparency in reporting. For further information on Nature Portfolio policies, see our Editorial Policies and the Editorial Policy Checklist.

Statistics

For all statistical analyses, confirm that the following items are present in the figure legend, table legend, main text, or Methods section.

n/a	Confirmed	
☒	☐	The exact sample size (*n*) for each experimental group/condition, given as a discrete number and unit of measurement
☒	☐	A statement on whether measurements were taken from distinct samples or whether the same sample was measured repeatedly
☒	☐	The statistical test(s) used AND whether they are one- or two-sided<br>*Only common tests should be described solely by name; describe more complex techniques in the Methods section.*
☒	☐	A description of all covariates tested
☒	☐	A description of any assumptions or corrections, such as tests of normality and adjustment for multiple comparisons
☒	☐	A full description of the statistical parameters including central tendency (e.g. means) or other basic estimates (e.g. regression coefficient) AND variation (e.g. standard deviation) or associated estimates of uncertainty (e.g. confidence intervals)
☒	☐	For null hypothesis testing, the test statistic (e.g. *F*, *t*, *r*) with confidence intervals, effect sizes, degrees of freedom and *P* value noted<br>*Give P values as exact values whenever suitable.*
☒	☐	For Bayesian analysis, information on the choice of priors and Markov chain Monte Carlo settings
☒	☐	For hierarchical and complex designs, identification of the appropriate level for tests and full reporting of outcomes
☒	☐	Estimates of effect sizes (e.g. Cohen's *d*, Pearson's *r*), indicating how they were calculated

Our web collection on statistics for biologists contains articles on many of the points above.

Software and code

Policy information about availability of computer code

Data collection	EPU 3.10.0
Data analysis	RELION 5.0, cryoSPARC v4.3.0, Warp 1.0.9, PHENIX 1.21.2-5419, COOT 0.8.9.2, UCSF ChimeraX 1.9, UCSF Chimera 1.19, PyMOL 2.5.2, PyMOL 3.1.3, xiVIEW 1.2.0, Jalview 2.11.4.1, XiSearch 1.8.6, ProteoWizard 3, XiFDR 2.3.5

For manuscripts utilizing custom algorithms or software that are central to the research but not yet described in published literature, software must be made available to editors and reviewers. We strongly encourage code deposition in a community repository (e.g. GitHub). See the Nature Portfolio guidelines for submitting code & software for further information.

Data

Policy information about availability of data

All manuscripts must include a data availability statement. This statement should provide the following information, where applicable:

- Accession codes, unique identifiers, or web links for publicly available datasets
- A description of any restrictions on data availability
- For clinical datasets or third party data, please ensure that the statement adheres to our policy

The cryo-EM reconstructions and final models were deposited with the EMDB under accession code EMD-52440 and the PDB under accession code 9HVO for the EC-RECQL5 complex, EMD-52443 (overall), EMD-52441 (SPT6-stalk), EMD-52442 (PAF) and PDB 9HVQ for the EC*-RECQL5 complex, and EMD-52449 and PDB

9HWG for the EC-TCR-RECQL5 complex. Mass Spectrometry Data is available at PRIDE PXD062042.
Following previously published PDB models were used in this study: 7B0Y, 6GMH, 8A3Y, 8A40, 8B3D

Research involving human participants, their data, or biological material

Policy information about studies with human participants or human data. See also policy information about sex, gender (identity/presentation), and sexual orientation and race, ethnicity and racism.

Reporting on sex and gender	N/A
Reporting on race, ethnicity, or other socially relevant groupings	N/A
Population characteristics	N/A
Recruitment	N/A
Ethics oversight	N/A

Note that full information on the approval of the study protocol must also be provided in the manuscript.

Field-specific reporting

Please select the one below that is the best fit for your research. If you are not sure, read the appropriate sections before making your selection.

☒ Life sciences ☐ Behavioural & social sciences ☐ Ecological, evolutionary & environmental sciences

For a reference copy of the document with all sections, see nature.com/documents/nr-reporting-summary-flat.pdf

Life sciences study design

All studies must disclose on these points even when the disclosure is negative.

Sample size	No statistical methods were used to predetermine the sample size. Cryo-EM data were collected to ensure an adequate number of particles for classification and refinement, aiming to achieve an overall resolution of < 3.5 angstroms, which allows the identification of amino acid side chains and model building. Our structures are all at or beyond 3.5 angstrom
Data exclusions	No data were excluded from the analyses.
Replication	All biochemical experiments were repeated in triplicates and attempts of replication were all successful.
Randomization	For 3D refinement the cryo-EM data were split randomly into two halves for gold-standard FSC determination
Blinding	Blinding is not relevant for these types of experiments. Structural and biochemical experiments require precise handling and extensive knowledge of the sample for data processing and analysis.

Reporting for specific materials, systems and methods

We require information from authors about some types of materials, experimental systems and methods used in many studies. Here, indicate whether each material, system or method listed is relevant to your study. If you are not sure if a list item applies to your research, read the appropriate section before selecting a response.

Materials & experimental systems

n/a	Involved in the study	
☐	☒	Antibodies
☐	☒	Eukaryotic cell lines
☒	☐	Palaeontology and archaeology
☒	☐	Animals and other organisms
☒	☐	Clinical data
☒	☐	Dual use research of concern
☒	☐	Plants

Methods

n/a	Involved in the study	
☒	☐	ChIP-seq
☒	☐	Flow cytometry
☒	☐	MRI-based neuroimaging

Antibodies

Antibodies used	F-12 anti-RPB1 antibody (1:1000 dilution, Santa Cruz Biotechnology, sc-55492), anti-CSB D-7 antibody (1:1000 dilution, Santa Cruz Biotechnology, sc-166042), HRP-conjugated anti-mouse secondary antibody (1:10,000 dilution, Proteintech, SA00001-1)
Validation	All the antibodies were validated by the manufacturers.<br>F12 is a mouse monoclonal antibody raised against amino acids 1-224 of Pol II RPB1 subunit of human origin, which has been validated for WB, IP, IF, IHC(P) and ELISA. Here it was used for Western blot.<br>CSB antibody is a mouse monoclonal antibody raised against amino acids 1-300 of CSB of human origin, which has been validated for WB, IP, IF, IHC(P) and ELISA. Here it was used for Western blot.

Eukaryotic cell lines

Policy information about cell lines and Sex and Gender in Research

Cell line source(s)	Hi5 cells: ThermoFisher, Gibco, Catalog number B85502<br>Sf9 cells: ThermoFisher, Gibco, Catalog Number 12659017<br>HEK293 cells: ATCC, Catalog Number CRL-1573
Authentication	Cell lines were guaranteed by the suppliers and therefore were not authenticated.
Mycoplasma contamination	Cell lines were not tested for mycoplasma contamination.
Commonly misidentified lines<br>(See ICLAC register)	No commonly misidentified cell lines were used.

Plants

Seed stocks	*Report on the source of all seed stocks or other plant material used. If applicable, state the seed stock centre and catalogue number. If plant specimens were collected from the field, describe the collection location, date and sampling procedures.*
Novel plant genotypes	*Describe the methods by which all novel plant genotypes were produced. This includes those generated by transgenic approaches, gene editing, chemical/radiation-based mutagenesis and hybridization. For transgenic lines, describe the transformation method, the number of independent lines analyzed and the generation upon which experiments were performed. For gene-edited lines, describe the editor used, the endogenous sequence targeted for editing, the targeting guide RNA sequence (if applicable) and how the editor was applied.*
Authentication	*Describe any authentication procedures for each seed stock used or novel genotype generated. Describe any experiments used to assess the effect of a mutation and, where applicable, how potential secondary effects (e.g. second site T-DNA insertions, mosiacism, off-target gene editing) were examined.*
