# Supplementary figures and images for: Structural basis of RECQL5-induced RNA polymerase II transcription braking and subsequent reactivation
Source: Nat Struct Mol Biol. 2025 Jul 7;32(9):1731–40. doi: 10.1038/s41594-025-01586-6 (PMC12440807; doi:10.1038/s41594-025-01586-6)

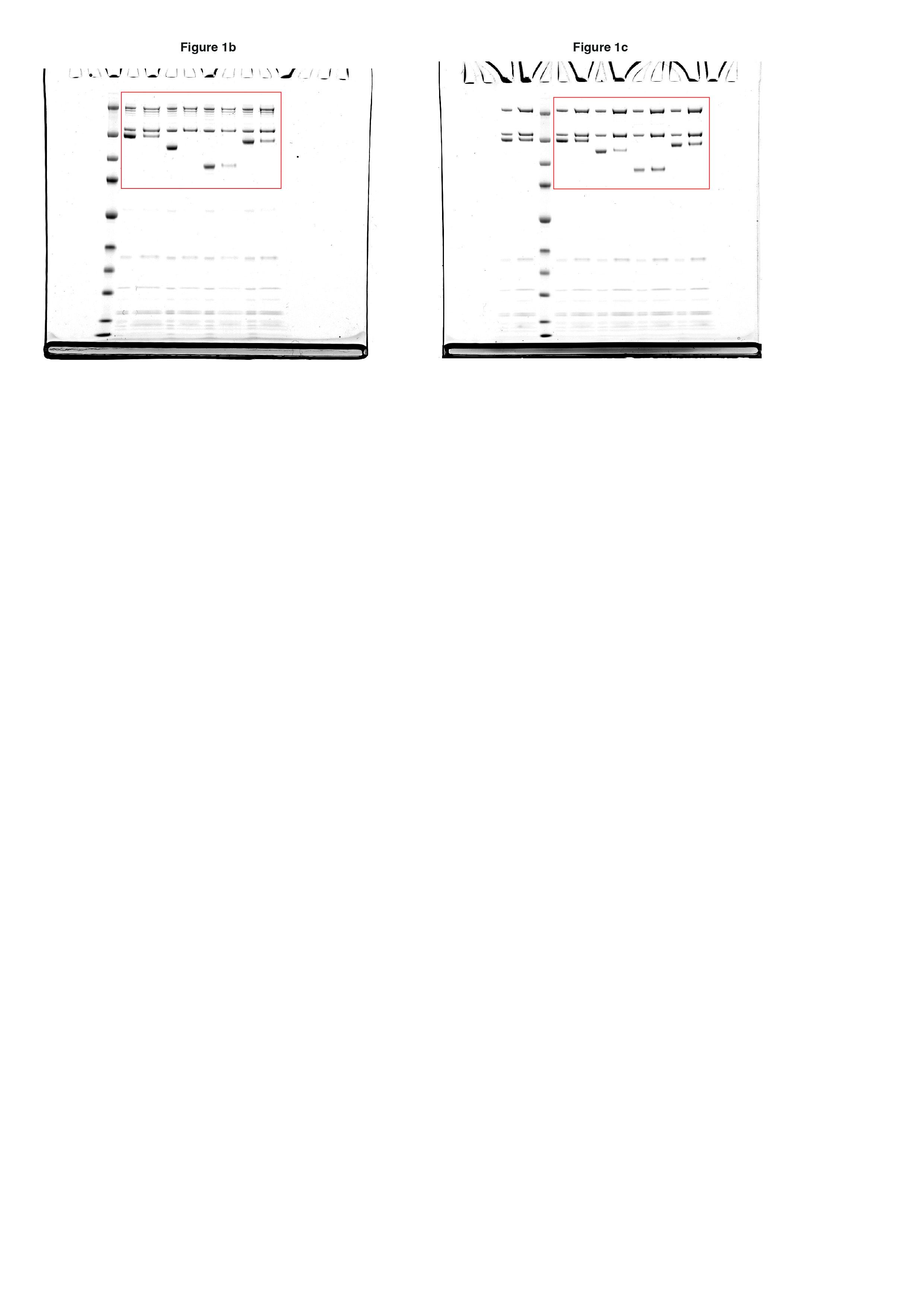

Supplement: Supplementary file 3 — Unprocessed gels. [file 41594_2025_1586_MOESM3_ESM.jpg]

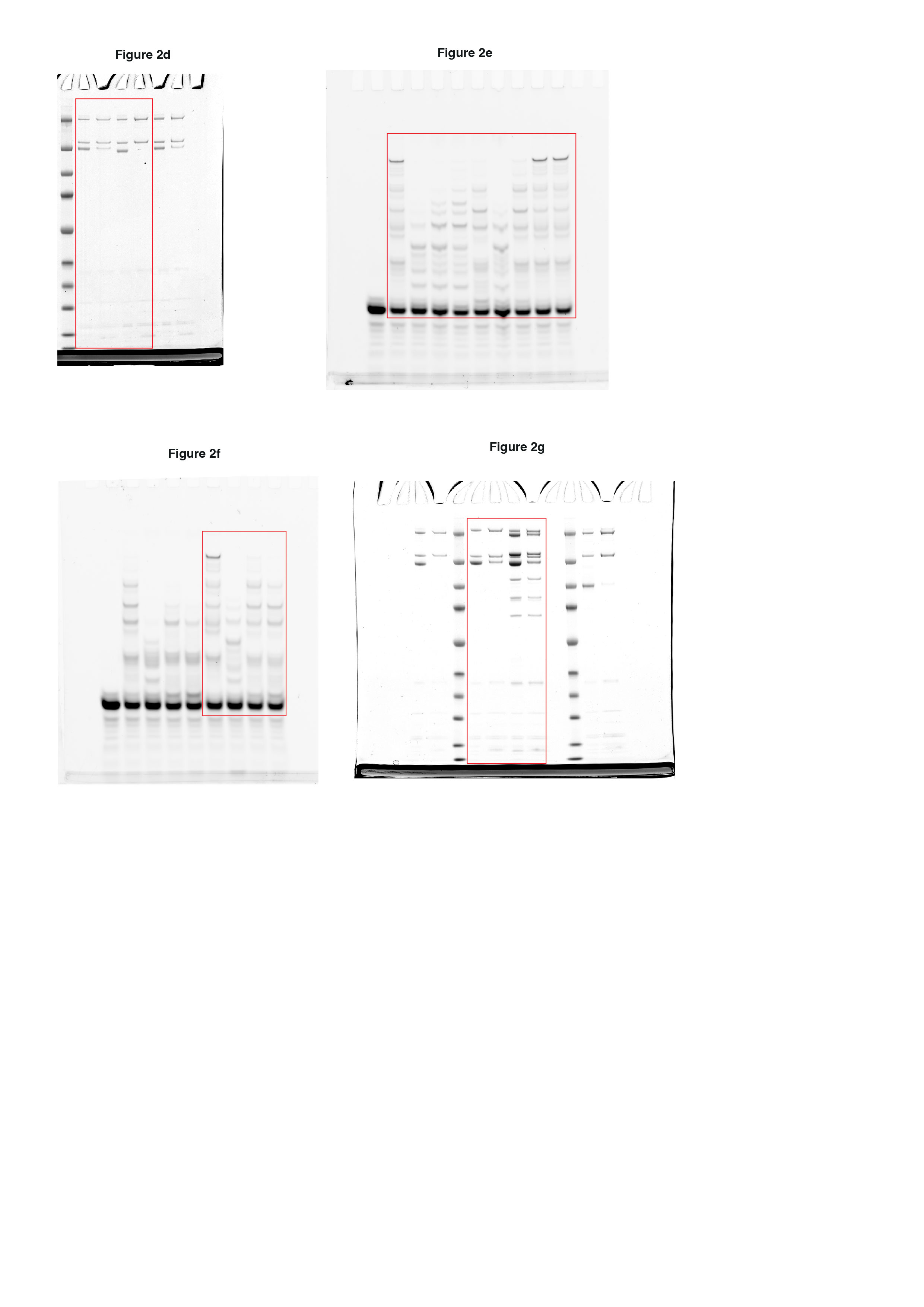

Supplement: Supplementary file 4 — Unprocessed gels. [file 41594_2025_1586_MOESM4_ESM.jpg]

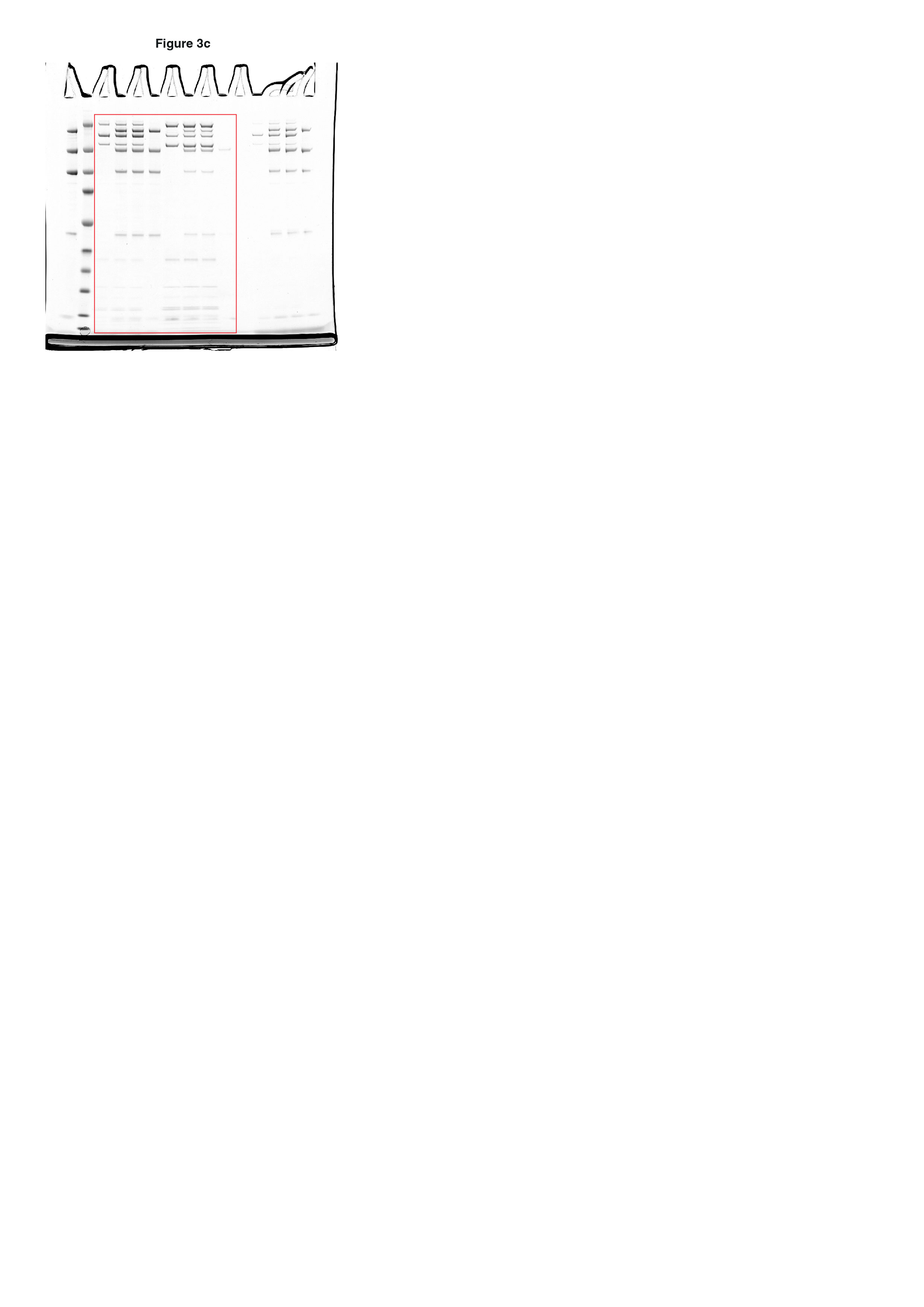

Supplement: Supplementary file 5 — Unprocessed gels. [file 41594_2025_1586_MOESM5_ESM.jpg]

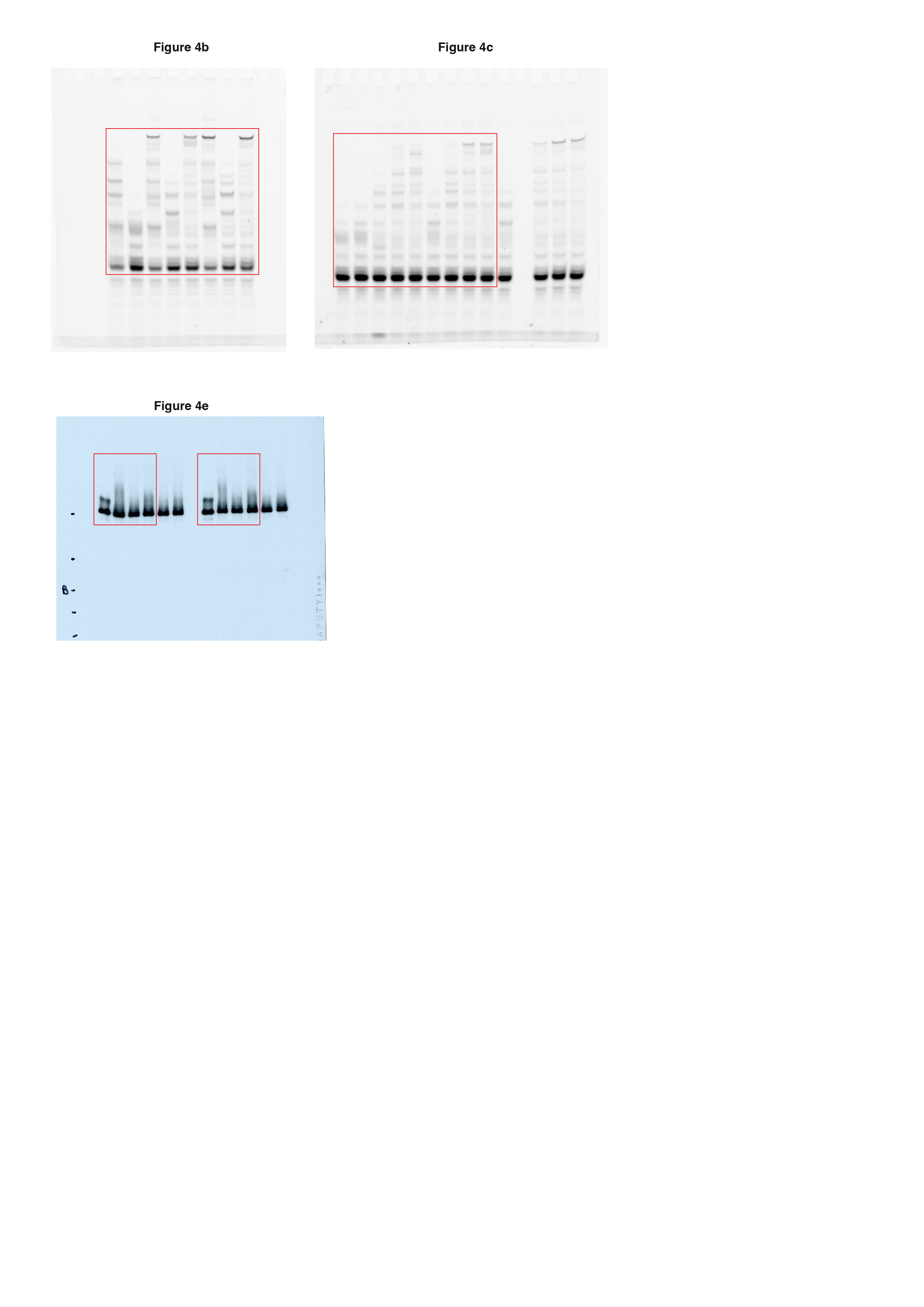

Supplement: Supplementary file 6 — Unprocessed gels and western blots. [file 41594_2025_1586_MOESM6_ESM.jpg]

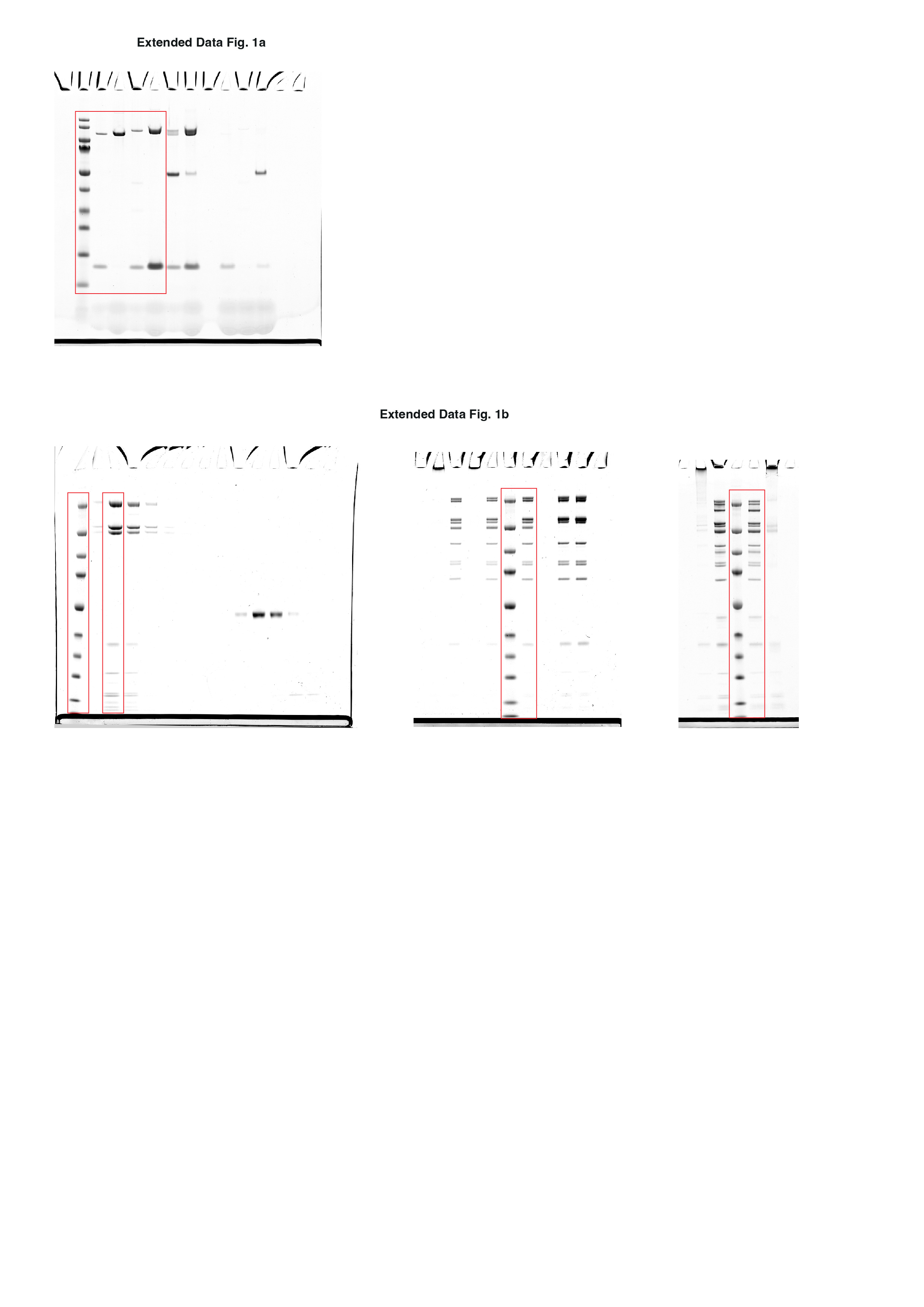

Supplement: Supplementary file 7 — Unprocessed gels. [file 41594_2025_1586_MOESM7_ESM.jpg]

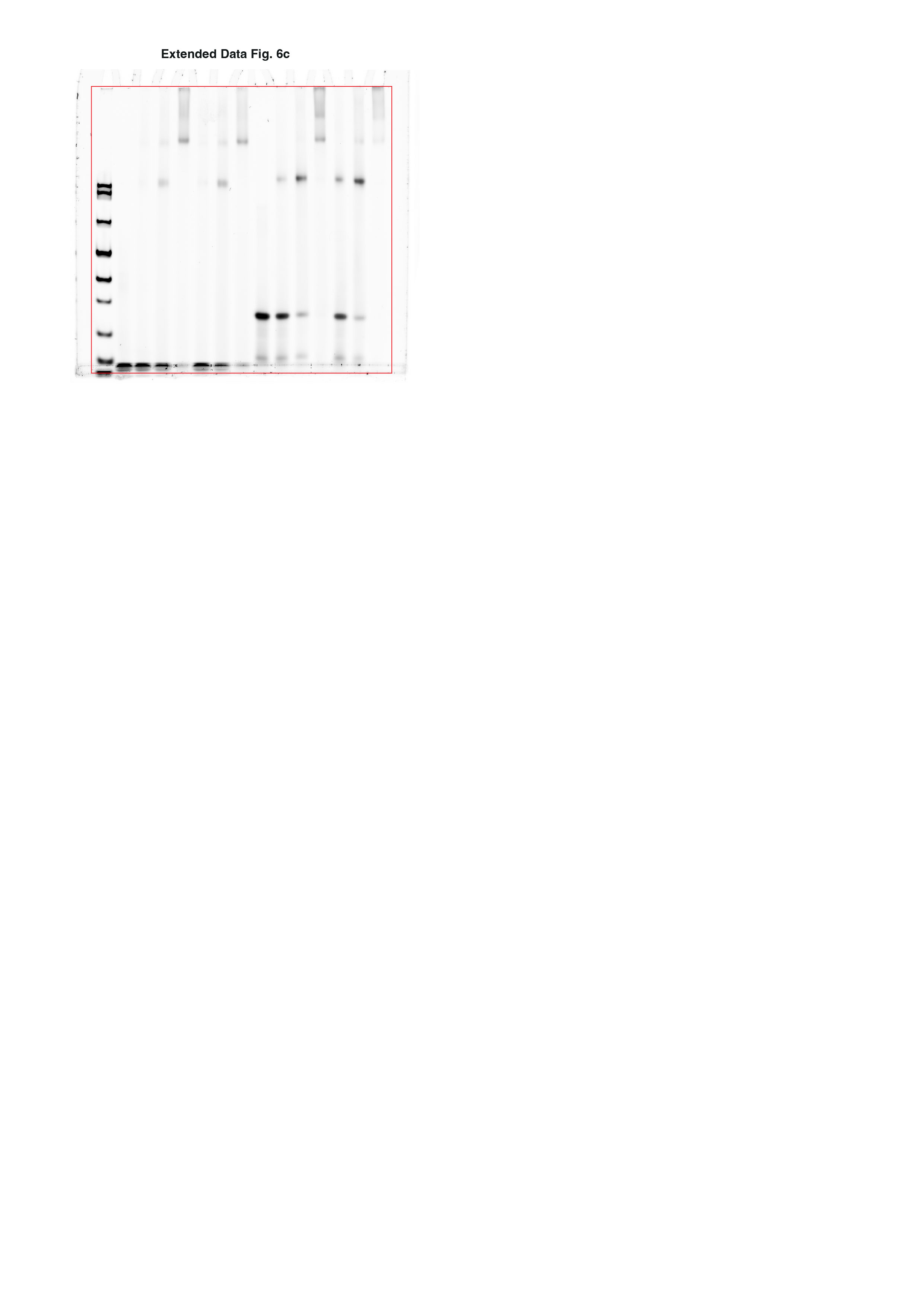

Supplement: Supplementary file 8 — Unprocessed gels. [file 41594_2025_1586_MOESM8_ESM.jpg]

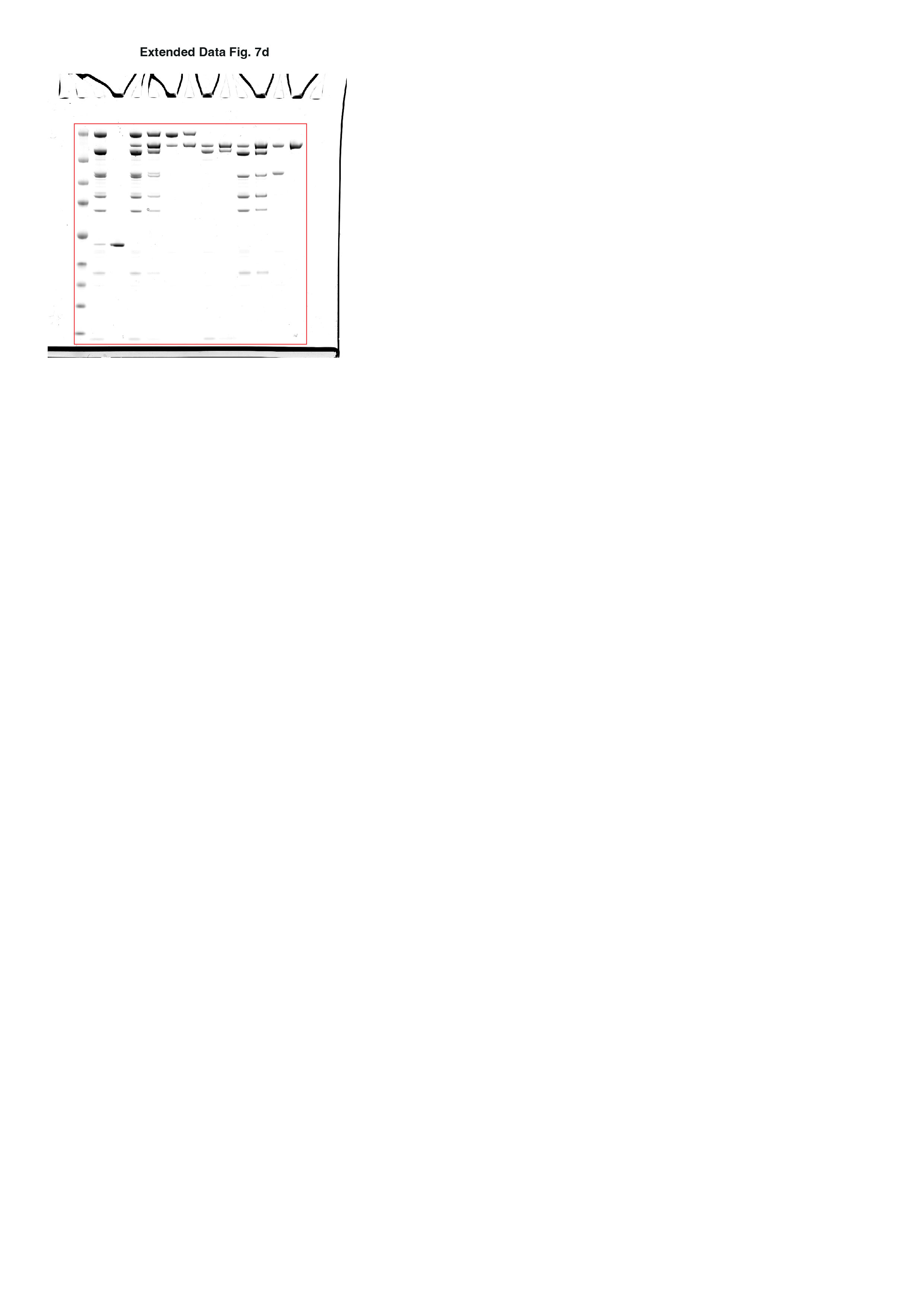

Supplement: Supplementary file 9 — Unprocessed gels. [file 41594_2025_1586_MOESM9_ESM.jpg]

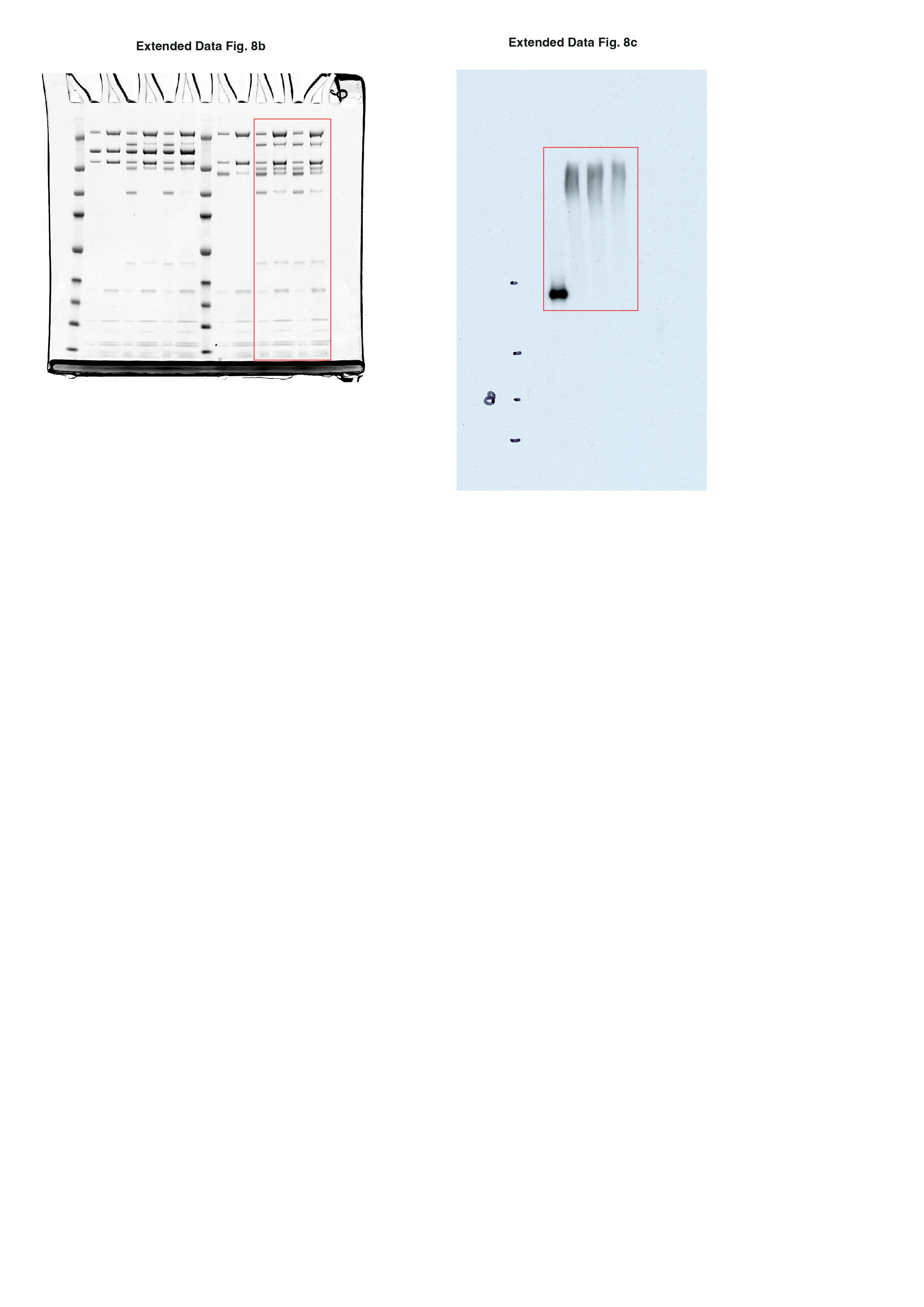

Supplement: Supplementary file 11 — Unprocessed gels and western blots. [file 41594_2025_1586_MOESM11_ESM.jpg]
